# Voluntary Medical Male Circumcision Programs Can Address Low HIV Testing and Counseling Usage and ART Enrollment among Young Men: Lessons from Lesotho

**DOI:** 10.1371/journal.pone.0083614

**Published:** 2014-05-06

**Authors:** Virgile Kikaya, Laura Skolnik, Macarena C. García, John Nkonyana, Kelly Curran, Tigistu Adamu Ashengo

**Affiliations:** 1 Jhpiego, Maseru, Lesotho; 2 Lesotho PEPFAR Program, U.S. Agency for International Development, Maseru, Lesotho; 3 Ministry of Health, Maseru, Lesotho; 4 Jhpiego, Baltimore, Maryland, United States of America; 5 Department of International Health, Johns Hopkins University Bloomberg School of Public Health, Baltimore, Maryland, United States of America; World Health Organization, Switzerland

## Abstract

**Background:**

Early diagnosis of HIV and treatment initiation at higher CD4 counts improves outcomes and reduces transmission. However, Lesotho is not realizing the full benefits of ART because of the low proportion of men tested (40%). Public sector VMMC services, which were launched in district hospitals in February 2012 by the Lesotho MOH supported by USAID/MCHIP, include HIV testing with referral to care and treatment. The objective of this study was to better understand the contribution of VMMC services to HIV diagnosis and treatment.

**Methods:**

VMMC clients diagnosed with HIV were traced after 6 months to ascertain whether they: (1) presented to the referral HIV center, (2) had a CD4 count done and (3) were enrolled on ART. Linkages between VMMC and HIV services were assessed by comparing the proportion of HIV-infected males referred from VMMC services with those from other hospital departments.

**Results:**

Between March and September 2012, 72 men presenting for VMMC services tested positive for HIV, representing 65% of the total male tests at the hospital; 45 of these men (62.5%) received an immediate CD4 count and went to the HIV referral site; 40 (89%) were eligible for treatment and initiated ART. 27 clients did not have a CD4 count due to stock-out of reagents. Individuals who did not receive a CD4 count on the same day did not return to the HIV center.

**Conclusion:**

All VMMC clients testing positive for HIV and receiving a CD4 count on the testing day began ART. Providing VMMC services in a district hospital offering the continuum of care could increase diagnoses and treatment uptake among men, but requires an investment in communication between VMMC and ART clinics. In high HIV prevalence settings, investing in PIMA CD4 devices at integrated VMMC clinics is likely to increase male ART enrolment.

## Introduction

HIV prevalence in Lesotho is among the highest in the world, with an estimated 23% of adults infected [1]. In Lesotho, an estimated 40% of men ages 15–49 have ever received an HIV test, compared to 68% of women [1]. Furthermore, Lesotho health survey data demonstrate that men are far less likely than women to know their HIV status or to have undergone an HIV test in the previous year. Studies have shown that men are more likely than women to start an antiretroviral therapy (ART) at a later stage with a more advanced HIV disease [2]–[3]. In addition, linkages between HIV testing and counseling (HTC) and ART services are generally weak, and clients tested in HTC programs often cannot be traced or followed up to ensure that they have enrolled in care and treatment. Yet early identification of HIV and enrollment in HIV care and treatment has been proven not only to improve health outcomes for HIV-infected individuals, but also to reduce HIV transmission.

In March 2007, WHO and UNAIDS issued guidance urging countries with high HIV prevalence and low male circumcision rates to incorporate male circumcision into their comprehensive HIV prevention programs. This recommendation was based on three randomized controlled trials that determined unequivocally that voluntary medical male circumcision (VMMC) reduces female to male HIV transmission by approximately 60% [4]–[6]. Post-trial surveillance also suggests that risk compensation has not been a problem in the clinical trial sites and that the population-level prevention benefit of VMMC has increased with time [7], [8]. Mathematical modeling studies suggest that scaling up VMMC to reach 80% coverage in 13 priority countries by 2015 could avert 3.36 million HIV infections by 2025 [9]. VMMC services are designed to be part of a comprehensive package that includes HTC, STI screening, condom promotion and health education. In countries such as Lesotho, where rates of HIV testing and ART enrollment are low especially among men, it is important to consider whether VMMC services could be an effective strategy to attract men to HTC and link them to care and treatment.

The VMMC program in Lesotho is implemented by the Lesotho Ministry of Health (MOH) with support from the Maternal and Child Health Integrated Program (MCHIP) led by Jhpiego and funded by the U.S. Agency for International Development (USAID). Consistent with the Lesotho National Health Strategic Plan, integrated public-sector VMMC services were launched in district hospitals in February 2012. These services are provided at no cost for all male clients presenting at VMMC clinics.

In the first 10 months of operation, it became clear that men in Lesotho were highly motivated to seek VMMC services. Between February and December 2012, more than 9,500 men (of whom 75% were between the ages of 15 and 24) were medically circumcised at one of six district hospitals in Lesotho, compared with only 400 men in the year before the launch of this national program [10]. Furthermore, 97% of clients presenting for VMMC services agreed to HIV testing; 5% of these men tested positive for HIV. Based on this performance, program implementers decided to retrospectively conduct secondary analysis of data collected at one hospital to further explore the linkages between VMMC, HTC and ART enrollment. In this study, we collected data on VMMC clients who tested HIV-positive in order to determine the outcome in terms of enrolment in HIV treatment and care.

## Methods

### Methods

Data were drawn from Lesotho's first public facility to be equipped to provide VMMC: Mafeteng government district hospital, located about 80 km from Maseru.

The number of clients accessing HTC and the percentage of positive results were extracted for all clients presenting for VMMC services between February and December 2012. HTC hospital statistics were reviewed to quantify the percentage of males 15–49 who tested at the VMMC clinic in comparison to other hospital department. VMMC clients testing positive for HIV were traced to ascertain whether they (1) presented at the ART clinic, (2) had received a CD4 count and (3) enrolled in treatment. Missed clients were followed through phone calls. Data were collected from the ART clinic in an effort to retrieve HIV-positive clients who enrolled in care and treatment. All data were collected from the hospital registers by the counselor in charge.

### Ethics statement

This work has a non-human subjects research determination notice from the Johns Hopkins School of Public Health IRB. The Ministry of Health of Lesotho does not require IRB review for secondary program data analysis as long as data are not identifiable.

All VMMC clients provided written informed consent before undergoing HIV testing as well as VMMC procedures as per national guidelines. As VMMC services are integrated into hospital services, all patient files are kept on site and only accessed by hospital providers. However, there was no separate informed consent collected specifically for this review. This research was waived by the IRB as it involved secondary data analysis of a pre-existing, de-identified/de-linked, not publicly available data set, and as investigators were not involved in the original data collection.

## Results

Males tested for HIV through VMMC services represented an important proportion of all males who were tested at Mafeteng district hospital from February to December 2012. Of the 2,941 males, ages 15–49, tested at Mafeteng hospital, 1,906 (65%) were tested at the VMMC clinic. Four hundred forty six (446) males tested HIV-positive at the hospital of which 72 (16%) were tested at the VMMC site.

Demographic information on VMMC clients who tested HIV-positive is included in table 1. Approximately 80% of VMMC clients who tested HIV-positive reside in Mafeteng district followed by 17% in Maseru district. There were about equal numbers of single and married clients, 47% and 49% respectively. Half of the clients were employed.

**Table 1:** Demographics of clients who tested HIV-positive at the Mafeteng VMMC site (n = 72).

	n	%
***District of origin***		
**Mafeteng**	57	(79.1%)
**Maseru**	12	(16.7%)
**Thaba Tseka**	1	(1.4%)
**Mohales'hoek**	1	(1.4%)
**Berea**	1	(1.4%)
***Marital status***		
**Single**	34	(47.2%)
**Married**	35	(48.6%)
**Divorced**	2	(2.8%)
**Widowed**	1	(1.4%)
***Employment status***		
**Employed**	36	(50.0%)
**Unemployed** *	36	(50.0%)

* *Includes students*.

**Figure 1:**
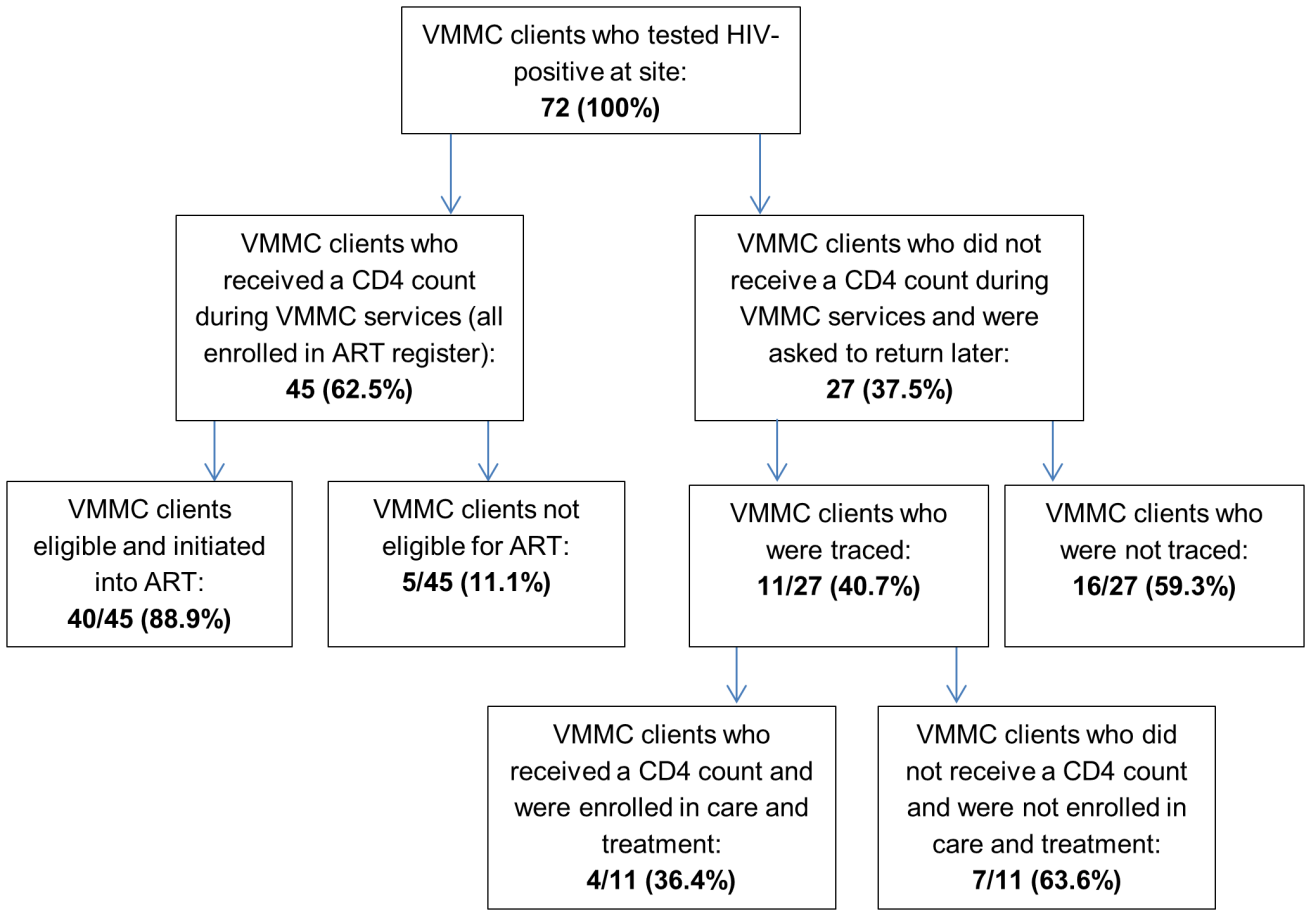
Flow chart describing ART enrolment outcome of VMMC clients who tested HIV-positive. All clients who received a CD4 count and who were eligible while seeking VMMC services were initiated on ART. Among the 27 clients who did not receive a CD4 count while seeking VMMC services, only four were enrolled in treatment.

The 72 VMMC clients testing positive were referred to the ART clinic for a baseline CD4 count (Figure 1). Forty-five (62.5%) of these clients received their CD4 count on the same day as being tested for HIV and receiving a positive result. The remaining 27 clients were unable to receive an immediate CD4 count due to a stock-out of reagents at Mafeteng district hospital between July and September 2012. These clients were referred to other district hospitals for the CD4 count or were requested to return at a later date. The mean CD4 count result was 302 (195–685). Among the 45 clients who received a CD4 count, 40 (88%) were eligible for treatment (i.e., CD4 count less than 350, as per Lesotho national guidelines). All clients who were eligible for treatment were enrolled and followed up. Figure 1 presents the algorithm for VMMC clients who tested HIV-positive.

The 27 clients who did not receive a CD4 count the same day as HIV diagnosis did not return to Mafeteng hospital for this service at a later date, as per ART register review. These clients were traced through phone calls. Sixteen could not be contacted because their phone numbers were not available. Among the 11 clients contacted, four reported that they received a CD4 count elsewhere and had enrolled in treatment; seven reported that they had not received a CD4 count.

## Discussion

The 2012 WHO strategic HTC program framework emphasizes the importance of ensuring linkages between HTC programs and prevention, treatment, care and support services [11]. VMMC services in Lesotho, an evidence-based biomedical intervention, include HIV testing as well as an increased focus on ensuring linkages with care and treatment as part of the package of services that are offered to clients.

The HIV continuum of care model provides a framework to identify issues and opportunities for improving HIV services delivery [12]. The two first steps of the model are HIV diagnosis and linkages to care and treatment. VMMC services implemented in a hospital setting provide an opportunity to effectively increase HIV testing among Basotho men and to ensure that linkages to HIV care and treatment are strengthened.

The provision of VMMC services in Lesotho has contributed to an increasing number of men who know their HIV status. As per the data reviewed at Mafeteng hospital, 65% of men who tested for HIV were tested at the VMMC clinic. In Lesotho, this contribution to testing services is of tremendous importance as the 2009 DHS suggests that almost 60% of men have never tested compared to 30% for women (age 15–59) [1]. In Lesotho, VMMC services represent a non-negligible opportunity to get men to know their status.

A number of studies are exploring how newly HIV diagnosed individuals are linked to care and treatment. Two studies discussed by Gardner et al. show that 73% and 64% of a cohort of newly identified HIV-positive individuals were enrolled in HIV care and treatment at one year and three months after diagnosis, respectively [13]. The same study showed that approximately 75% of a cohort of individuals who tested HIV-positive were successfully linked to HIV care within six to 12 months, and 80% to 90% of the cohort after three to five years. At Mafeteng hospital, an estimated 68% of all HIV-positive VMMC clients were linked to care at the time of the study. Several factors can influence the proportion of individuals who are linked to care and treatment. WHO recommends that linkage to care and treatment be more rigorously evaluated [14] and that good practices that can improve such linkages be identified. According to WHO recommendations, these good practices include but are not limited to providing on-site or immediate CD4 testing with same-day results; assisting with transport if the ART site is far from the HTC site; and involving community outreach workers to identify the people lost to follow-up [14]. This study identified additional elements that might influence linkages in the context of VMMC services being implemented in a hospital setting in Lesotho, including availability of CD4 count laboratory tests, which is consistent with WHO recommendations. However, additional elements such as the attitude of services providers, the routine review of information and weaknesses in the national supply chain were identified as elements influencing the effectiveness of linkages to care and treatment.

### CD4 count on site

The findings from the secondary analysis of programmatic data strongly suggest that clients testing positive for HIV should be offered a CD4 count immediately following diagnosis. This study shows that when this occurs, clients are more likely to enroll in HIV care and treatment. Receiving a CD4 count immediately provides an opportunity for clients to be counseled on HIV care and treatment, to be enrolled into services and increases adherence outcomes. VMMC clients who did not get a CD4 count in the VMMC clinic after HIV-positive diagnosis were less likely to either return or to get a CD4 count elsewhere. This finding demonstrates the importance of having a CD4 count available at the point of care, preferably within the VMMC clinic. Given that VMMC clients return for follow-up care after 48 hours and again within seven days from the date of procedure, VMMC services provide an opportunity to ensure that men are effectively linked to HIV treatment and care services—especially in Lesotho, where VMMC services are offered and integrated within hospital services. In Mafeteng, the laboratory is informed of routine days for VMMC services and VMMC hospital-based campaigns and collaborates closely with VMMC providers. Ensuring that other hospital units are aware and oriented on VMMC services and that they recognize VMMC service provision as a catalyst for increased HIV testing among Basotho men is essential to strengthen linkages to HIV prevention, care and treatment.

### Hospital staff

VMMC services in Lesotho are delivered by a team of medical doctors, nurses and HTC counselors. At Mafeteng hospital, the success of the referral and linkage program is due largely to having hospital staff trained in both ART and VMMC services. Nurses draw blood for patients' CD4 count at the VMMC site and take it to the laboratory. The VMMC counselor, who works at both the VMMC site and the ART clinic, has experience in countrywide HTC campaigns and proactively ensures that nurses from the ART clinic are available to collect blood samples on VMMC clinic days. The counselor, when necessary will ensure physical referral (escorting) to the ART clinic. VMMC providers also foster positive communication with the laboratory and other services to ensure on-time CD4 count results are available to VMMC clients.

### Monitoring and evaluation of VMMC clients' clinic data

VMMC providers at Mafeteng hospital maintain detailed data statistics on clients' HIV testing and diagnosis. The records are then integrated with hospital data. For this study, the VMMC counselor cross-referenced data with information at the ART clinic to identify VMMC clients who were not enrolled in HIV care and treatment and had not yet returned. Counselors then attempted to contact these clients. This review highlights the need to ensure routine data review and analysis of the VMMC and the ART clinic data sets.

### Ensuring supply for HIV commodities

The review of VMMC program data identified stock-outs of essential HIV-related commodities, as a challenge to the provision of services to clients attending VMMC programs. In Lesotho, because of a stock-out of CD4 reagents, dozens of clients who tested positive for HIV through the VMMC program were unable to receive a CD4 count at the time of their visit and did not come back for it later. This fact highlights the need to optimize linkages between VMMC services and HIV care and treatment by also strengthening supply chain management systems.

## Conclusion

All VMMC clients who tested positive for HIV and who received a CD4 count on the day of diagnosis were initiated on ART. The provision of VMMC within an integrated service delivery setting such as a district hospital has contributed to the increase of HIV testing among men in Lesotho. Moreover, the VMMC program in Lesotho has the potential to increase HIV-positive men's enrollment in ART given that linkages with HIV care and treatment have been strengthened. The successful linkages between VMMC clinic clients to HIV care and treatment services provides an important opportunity given Lesotho's high HIV prevalence and low rates of HIV testing among men.

The Mafeteng district hospital model, which consists of integrated services and effective hospital department communications, provides a successful framework to be replicated elsewhere. Study findings serve as a foundation for further analysis of the potential of VMMC services as an entry point to care and treatment for HIV-positive men in other hospital settings.

However, improving VMMC–ART linkages will require an investment in follow-up with clients and communication between VMMC and ART clinics. In high HIV prevalence settings, investing in PIMA CD4 devices at integrated VMMC clinics will also likely increase male enrolment in care and treatment services, as was evident in this study.
